# Efficiency of δ-Tocopherol in Inhibiting Lipid Oxidation in Emulsions: Effects of Surfactant Charge and of Surfactant Concentration

**DOI:** 10.3390/antiox12061158

**Published:** 2023-05-26

**Authors:** Tamara Martínez-Senra, Sonia Losada-Barreiro, Carlos Bravo-Díaz

**Affiliations:** Departamento Química-Física, Facultad de Química, Universidade de Vigo, 36310 Vigo, Spaincbravo@uvigo.es (C.B.-D.)

**Keywords:** charged interfaces, tocopherol, emulsions, partition constant, efficiency, soybean oil

## Abstract

Charged interfaces may play an important role in the fate of chemical reactions. Alterations in, for instance, the interfacial acidity of emulsions induced by the charge of the surfactant head group and associated counterions may change the ionization status of antioxidants, modifying their effective concentrations. The chemical reactivity between interfacial reactants and charged species of opposite charge (protons, metallic ions, etc.) is usually interpreted in terms of pseudophase ion-exchange models, treating the distribution of charged species in terms of partitioning and ion exchange. Here, we focus on analyzing the effects of charged interfaces on the oxidative stability of soybean oil-in-water (o/w) emulsions prepared with anionic (sodium dodecyl sulfate, SDS), cationic (cetyltrimethylammonium bromide, CTAB) and neutral (Tween 20) surfactants, and some of their mixtures, in the presence and absence of δ-tocopherol (δ-TOC). We have also determined the effective concentrations of δ-TOC in the oil, interfacial and aqueous regions of the intact emulsions. In the absence of δ-TOC, the relative oxidative stability order was CTAB < TW20 ~ TW20/CTAB < SDS. Surprisingly, upon the addition of δ-TOC, the relative order was SDS ≈ TW20 << TW20/CTAB < CTAB. These apparently surprising results can be rationalized in terms of the nice correlation that exists between the relative oxidative stability and the effective interfacial concentrations of δ-TOC in the various emulsions. The results emphasize the importance of considering the effective interfacial concentrations of antioxidants in interpreting their relative efficiency in emulsions.

## 1. Introduction

Oil-in-water (o/w) food emulsions are complex colloidal entities whose main constituents are lipid molecules, mostly located in the core of droplets dispersed in an aqueous fluid, and where surfactants and proteins are embedded in the interfacial region to maintain the physical stability of the emulsified system [1,2]. When prepared in contact with oxygen, the unsaturated lipids undergo oxidation, and the oxidation products can lead to the deterioration of the sensory properties and nutritional values of the emulsions during production, transport and storage, leading to the potential rejection of the product by consumers [3,4].

The food industry employs naturally occurring surfactants, such as egg lecithins (to prepare, for instance, mayonnaise), and/or synthetic surfactants, such as sorbitan esters and their ethoxylates, to kinetically stabilize food emulsions [5,6,7]. In addition to their central role in maintaining the physical stability of emulsions, surfactants may also affect the kinetics of lipid oxidation. For instance, an increase in the surfactant concentration increases the interfacial volume, leading to an effective decrease in the effective concentration of reactants. The electrical charge of the emulsifier is also one of the critical factors that may affect the fate of the lipid oxidation reaction because it can affect the effective concentration of transition metals and antioxidants through attractive or repulsive forces or by modifying the ionization status of the antioxidants [3,4,5,8].

**Figure 1:**
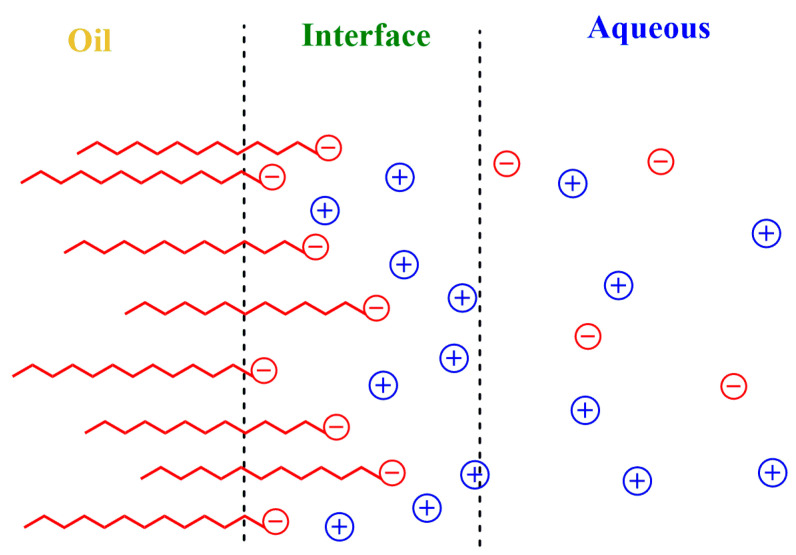
Schematic representation of the Gouy–Chapman diffuse layer of a small section of an emulsion droplet. The electrical potential decreases with distance from the oil core. The interfacial region is a transition region with properties between those of the aqueous and oil phases and contains the ionic head groups of the surfactants, some oil, water, a fraction of the counterions and, eventually, some co-ions.

Surfactants readily (and rapidly) adsorb at the oil–water interface, forming an interfacial layer of a few nanometers whose limits are difficult to determine precisely [9,10]. The interfacial layer contains the ionic head groups of the surfactants, some oil, water and a fraction of the counterions. It is, therefore, an anisotropic region with properties ranging from those of bulk water to those of the bulk oil. Thermal motion creates a diffuse electrical double layer that extends to the water phase, as shown in Figure 1, and contains the remaining counterions and other emulsion components [11]. Because of the thermal motion and the restriction-less movement between regions, ions and other molecules can be exchanged, having significant effects on lipid oxidation reactions, on their inhibition by antioxidants and, in general, on chemical reactivity [12,13,14,15,16].

Most literature reports that analyzed the role of the interfacial charge in the control of lipid oxidation, both in the absence and in the presence of antioxidants, have been focused on evaluating its effects on the location of prooxidant metals, but the results are not conclusive. Some reports indicate that negatively charged (cationic) o/w emulsions show higher oxidative stability compared to that of emulsions prepared with anionic or neutral surfactants, an effect commonly attributed to the repulsion between the charge of the surfactant head group and potential prooxidant metal ions. However, other reports indicate that cationic surfactants decrease the oxidative stability of emulsions because they accelerate the decomposition of hydroperoxides, but anionic ones do not and thus are more stable [17].

Yi et al. [8] investigated the effects of the emulsifier charge on the oxidative stability of o/w emulsions, concluding that the oxidative stability of emulsions prepared with Tween 20 depends on the concentrations of FeCl_3_ and/or FeCl_2_. However, the same authors reported that the oxidative stability in samples containing CTAB increased upon increasing [FeCl_3_] and [FeCl_2_] up to 0.5 mM, but a further increase in the metal concentration caused the oxidative stability to decrease, leading the authors to conclude that CTAB “acts differently” during lipid oxidation compared to SDS and Tween 20 surfactants, probably because CTAB affects the formation of hydroxyperoxides.

Osborn et al. [18] investigated the effects of iron, acidity and the hydrophobicity of antioxidants (α-tocopherol, gallic acid and quercetin), suggesting that the physical location of phenolics affects their ability to influence lipid oxidation. Mei et al. investigated the effects of the charge status of both phenolic antioxidants and emulsion droplets on lipid oxidation rates. They reported that in SDS-stabilized emulsions, the rates of oxidation were higher than in neutral (Brij) emulsions, hypothesizing that such differences were related to differences in the partitioning of antioxidants and to increases in the prooxidant effects of Fe^2+^ ions [19,20]. Unfortunately, the authors did not quantify such effects.

The charge of the surfactant is not the only factor affecting chemical reactivity in emulsions [10,21,22,23]. The Hydrophilic–Lipophilic Balance (HLB) of the surfactant may also affect the fate of the lipid oxidation reaction because it can modify the distribution of relevant emulsion components, e.g., the effective concentrations of antioxidants and their orientation [21,24,25,26,27,28]. Stöckman et al. [29] investigated the partitioning of a series of hydroxybenzoic acids in different oil-in-water systems, concluding that the nature of the surfactant affects their partitioning. Losada et al. [30] investigated the effects of the HLB of neutral surfactants and their concentrations on the distributions of hydrophilic (gallic acid, GA) and hydrophobic (α-tocopherol, α-TOC) components in corn emulsions. They concluded that an increase in the HLB of the surfactant favors the incorporation of α-TOC into the interfacial region of the emulsion but has a negligible effect on the percentage of GA in that region. However, the HLB of the antioxidant has a significant effect on the fate of the lipid oxidation inhibition reaction because of the differential partitioning of the antioxidant in the various regions of the emulsion [27,31].

To gain further insights into the role of the charge and the HLB of surfactants in the efficiency of antioxidants in inhibiting lipid oxidation, we investigated the effects of the interfacial charge and of the surfactant concentration on the distribution and antioxidant efficiency of a natural antioxidant, δ-tocopherol. The location of antioxidants and their effective concentrations are central to interpreting their efficiency in inhibiting lipid peroxidation, and thus, our aim here is to contribute to the general understanding of how antioxidants and droplet charges affect the distributions of antioxidants and the lipid oxidation reaction. For this purpose, we prepared emulsions composed of stripped soybean oil and representative nonionic (Tween 20), anionic (sodium dodecyl sulfate, SDS) and cationic (cetyltrimethylammonium bromide, CTAB) surfactants, as well as some of their mixtures.

We have chosen δ-tocopherol as a model antioxidant because tocopherols are one of the most important nonpolar free-radical-scavenging antioxidants in foods and biological tissues, decreasing the rate of lipid oxidation by donating hydrogen to a fatty acid radical and forming relatively stable phenolic free radicals [32,33,34]. The tocopherol homologs (δ-, β-, γ- and α-tocopherols containing 1, 2, 2 and 3 methyl groups in the chromanol ring, respectively) have different antioxidant activities in o/w emulsions and bulk oils, usually attributed to their differences in polarity [33,35]. Huang and co-workers [34] reported that the more polar γ-tocopherol was a more effective antioxidant than α-tocopherol in corn o/w emulsions. It was also found that the antioxidant activity of the more polar δ-tocopherol was better than that of α-tocopherol in menhaden o/w emulsions, while in bulk menhaden oil, α-tocopherol was a more effective antioxidant than δ-tocopherol [36].

Since lipid oxidation and its inhibition by antioxidants may involve charged species (e.g., protons and/or metals), and their concentration at the interfacial region may modify the rates of the various reactions involved, it may be useful to have an understanding of the kinetic effects that one may expect to find when dealing with emulsions bearing charged interfaces. The main kinetic consequences are related to concentration and ion-exchange effects that arise from electrostatic interactions, and they are briefly described in the next section.

### An Overview of the General Effects of Charged Interfaces on Chemical Reactivity

Emulsions are thermodynamically unstable systems that need some energy input to be created [9,10]. They can be stabilized kinetically by adding surfactants, which adsorb at the oil–water interface, creating an interfacial region with a thickness of several nanometers. An excess of the surfactant may spontaneously self-associate in the aqueous region, creating dynamic aggregates (micelles, reverse micelles, microemulsions) of various forms, depending on the surfactant structure and solution composition. All of them, including emulsions, have a common feature: they contain an interfacial region separating a polar bulk aqueous phase from an apolar, hydrocarbon-like region. When the surfactants employed are cationic or anionic, a fraction of the counterions remain in the interfacial region, and the thermal motion creates a diffuse electrical double layer, called the Gouy–Chapman layer, which extends to the aqueous phase and contains the remaining counterions, as shown in Figure 1.

**Figure 2:**
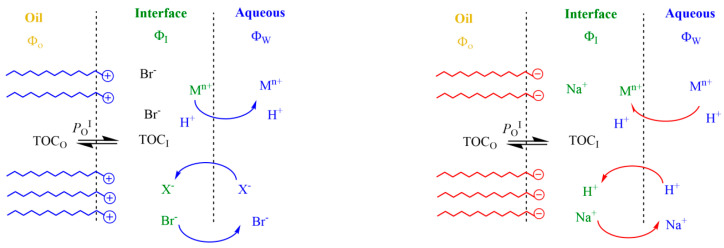
Main effects of charged interfaces on the distribution of reactive ions. Cationic emulsions (**left**) repel positively charged species, decreasing their effective concentrations in the interfacial region, while they attract negatively charged species. In contrast, anionic emulsions (**right**) attract positively charged species (protons, metals, etc.), exchanging with them at the same time that negatively charged species are repelled. In both cases, the exchange of ions takes place until reaching an equilibrium so that the delicate balance between hydrophobic and electrostatic forces maintain the physical structure of the emulsion.

The electrostatic attractive/repulsive forces generated due to the presence of interfacial charges modify the interfacial concentrations of reactive ions such as H^+^ or metal ions M^n+^ (Fe^2+^, Cu^2+^, etc.) because charged emulsions act as ion-exchangers, as illustrated in Figure 2. For instance, in anionic SDS interfaces, H^+^ and Na^+^ (or any other monovalent metal ion M^+^) ions are exchanged so that the counter-cation concentrations in the interfacial region are in large excess over the anion concentrations. As a result, interfacial H^+^ concentrations in emulsions are significantly different (1–2 orders of magnitude) from those in the bulk solution [13,37,38,39]. Ion exchange is commonly described by an empirical ion-exchange constant, as given by Equation (1), where the subscripts I and W describe the interfacial and aqueous regions, respectively [17,32].

Under a given (fixed) set of experimental conditions, an equilibrium is established between ions located in the interfacial region of the emulsion (bounded ions) and the remaining ions in the aqueous phase. This two-site model has been proved experimentally in micellar systems by employing different experimental techniques that quantitatively measure either bound- or free-ion concentrations [40,41,42,43] and should also be applicable to emulsions because emulsion droplets and micelles are present simultaneously and typically exchange material at diffusion-controlled rates [21,44,45,46]. Thus, in emulsions formed with a surfactant with counterions X with charge Zx and containing other counterions Y with charge Zy, the equilibrium condition for each counterion requires that the electrochemical potentials of aqueous and bounded counterions be equal to each other, and the ion-exchange constant given by Equation (1) can be derived [13,15,43,47]. Relevant counterions for the lipid oxidation reaction and its inhibition by antioxidants are protons, prooxidant metals or OH^−^ ions, which can exchange with inert ions such as Na^+^ and Br^−^. When divalent or trivalent metals, M^n+^, are present, metals are also exchanged, and their distribution is defined by an empirical ion-exchange constant, as shown in Equation (2), where SD^−^ stands for the negatively charged dodecyl sulfate monomers. Note that, in these cases, two or more monovalent ions are exchanged with only one metal ion (depending on the metal charge), and the corresponding ion-exchange constants are usually larger than those of monovalent ions, according to the Hofmeister series [48].

(1) $${\mathrm{nH}}_{I}^{+}+M_{W}^{n+}\overset{K_{M}^{H}}{⇌}{\;\mathrm{nH}}_{W}^{+}{+M}_{I}^{n+}$$

(2) $${n(\mathrm{SD}}^{-}{\mathrm{Na}}^{+}{)}_{I}+M_{W}^{n+}\;\overset{K_{M}^{\mathrm{Na}}}{⇌}{\;n(\mathrm{SD}}^{-}{)}_{I}M_{I}^{n+}{+\mathrm{nNa}}_{W}^{+}$$

At cationic interfaces, the exchange is described by an empirical Donnan equilibrium constant between H^+^ and Br^−^ [41,42]. Within cationic interfaces, the interfacial H^+^ concentration may be significantly lower, by one to two orders of magnitude, than the bulk [H^+^]. Cationic interfaces may repel metallic ions, M^n+^, thus decreasing their effective interfacial concentrations, but may attract anions, as described in Equation (3). Cationic and zwitterionic emulsion interfaces selectively bind anions, typically following the Hofmeister series, as demonstrated by Gao et al. [13].

(3) $${n(\mathrm{CTA}}^{+}X^{-}{)}_{I}+Y_{W}^{n-}\;\overset{K_{Y}^{X}}{⇌}{\;n(\mathrm{CTA}}^{+}{)}_{I}Y_{I}^{n-}{+\mathrm{nX}}_{W}^{-}$$

Thus, charged interfaces not only affect the physical stability of emulsions [9] but also affect the kinetics of the reactions taking place therein by significantly altering the interfacial acidity (cationic surfactants reduce it, and anionic surfactants increase it) and/or modifying the charge status of antioxidants. In addition, charged interfaces modify some physical properties of antioxidants (for example, their acid–base equilibria), affecting their partitioning within the emulsion.

## 2. Materials and Methods

### 2.1. Materials

All chemicals were of the highest purity available (>95%) and used as received. Surfactants employed to kinetically stabilize the emulsions (polyoxyethylene (20) sorbitan monolaurate (Tween 20, TW20), Sodium dodecyl sulfate (SDS) and cetyltrimethylammonium bromide (CTAB)) were from Fluka, Buchs (Switzerland). Distilled and deionized water (conductivity < 0.1 µS cm^−1^) was used in all experiments. The acidity of the aqueous phase (pH = 3.5–6.0) was controlled by employing citric acid/citrate buffer (Acros Organics, Geel (Belgium), >99%, 0.04 M). Aqueous solutions of pH = 8.0 were prepared by employing phosphoric acid/sodium phosphate (Sigma-Aldrich, Darmstadt, Germany, 85%) and the necessary amounts of NaOH. Stock solutions of δ-TOC (~0.5 M) were prepared by dissolving the required amount of δ-TOC in a 1:1 (*v*/*v*) EtOH/BuOH mixture and were used immediately.

4-Hexadecylbenzenediazonium tetrafluoroborate, 16-ArN_2_BF_4_, was prepared from 4-hexadecylaniline (Aldrich, 97%) by diazotization with butyl nitrite in acidic solution, as described elsewhere [45], and was stored in the dark at a low temperature to minimize its spontaneous reaction with water vapor. Solutions of the coupling agent N-(1-Naphthyl)ethylenediamine (NED, Aldrich) were prepared in a 1:1 (*v*/*v*) BuOH:EtOH mixture to give [NED] = 0.02 M. Soybean oil was a generous gift from Aceites Abril (Ourense, Spain). The endogenous antioxidants in the oil were removed by washing it with a 0.5 M NaOH solution and passing it twice through an activated Al_2_O_3_ column. The absence of endogenous antioxidants was checked by HPLC according to standard procedures (IUPAC method 2.432).

### 2.2. Emulsion Preparation

First, 1:9 and 4:6 (*v*/*v*, V_T_ = 10 mL) soybean oil-in-water emulsions were prepared by mixing 1 mL of stripped soybean oil with 9 mL of an aqueous acid solution (0.04 M citrate buffer, pH 3.5) and a weighed amount of the surfactants. An aliquot of the 0.5 M δ-TOC stock solution was added to the oil before mixing with the aqueous surfactant solution so that the surfactant volume fraction in the emulsion, defined as Φ_I_ = V_surf_/V_emulsion_, varied from Φ_I_ = 0.005 to Φ_I_ = 0.04. Table 1 shows the percentage of each surfactant in the prepared emulsions and the corresponding HLB, calculated by using Equation (4), where x stands for the percentage (%) of the surfactant employed.

(4) $${\mathrm{HLB}=x\;\mathrm{HLB}}_{A}{+(1-x)\;\mathrm{HLB}}_{B}$$

Mixtures were stirred with the aid of a high-speed rotor (Polytron PT 1600 E) for at least 2 min, and the freshly prepared emulsions were transferred to thermostated cells with continuous magnetic stirring (distribution experiments) or orbital shaking (oxidation experiments). The stability of the emulsions was checked visually, and no phase separation or other instability phenomena (creaming, sedimentation, etc.) were observed within several (>5) days, a much longer time than that required to complete the kinetic experiments.

### 2.3. Interfacial Charge of Emulsions: ξ Potential

The values of ζ potential were determined by employing a particle electrophoresis instrument (Zetasizer Nanoseries Nano-ZS, Malvern Instruments, Worcestershire, UK). In a typical experiment, emulsions were diluted 1:10 (*v*/*v*) by employing an aqueous solution of the same pH as that employed in their preparation, and measurements were obtained in triplicate. Only the average values are displayed in Table 1.

### 2.4. Application of the Pseudophase Kinetic Model

**Figure 3:**
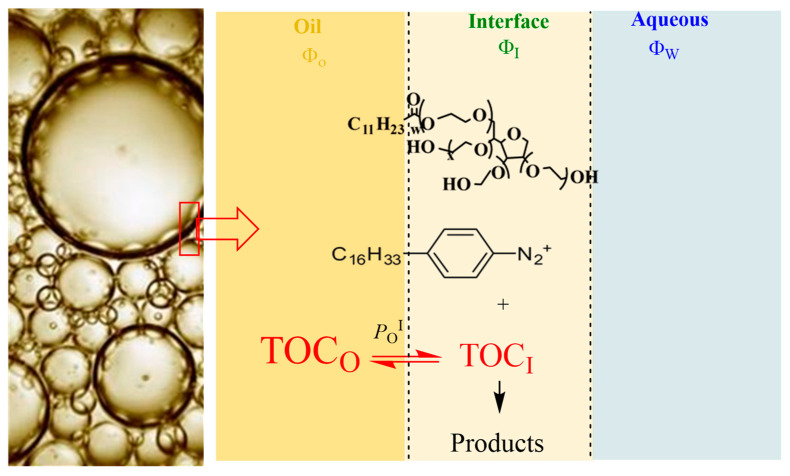
Distribution of a water-insoluble antioxidant AO (e.g., δ-TOC) in emulsions. Only the partition constant between the oil and the interfacial region is needed to describe its distribution; however, a second partition constant, that in the aqueous–interfacial region (*P*_w_^I^), is also necessary to describe the distribution of antioxidants with moderate hydrophobicity between the oil, interfacial and aqueous regions of emulsions.

The distribution of AOs and their interfacial concentrations need to be determined in the intact emulsions to avoid the disruption of the existing equilibria. For this purpose, a special kinetic protocol was employed (Figure 3). The method exploits the chemical reaction between a hydrophobic arenediazonium ion, 4-hexadecylbenzenediazonium, 16-ArN_2_^+^, and the antioxidant to determine the partition constants in the aqueous–interfacial, *P*_W_^I^, and oil–interfacial, *P*_O_^I^, regions of the emulsions [13,30,39,45,49,50,51]. The chemical probe 16-ArN_2_^+^ is located exclusively in the interfacial region of the emulsions, as shown in Figure 3, where it reacts with the antioxidant. The partition constant of a hydrophobic antioxidant such as δ-TOC, which is water-insoluble, in the oil–interfacial region, *P*_O_^I^, is defined by Equation (5) [52,53,54], and the mathematical relationship between the observed rate constant *k*_obs_ and the partition constant of hydrophobic antioxidants given by Equation (6) can be established. Details on the derivatization of Equation (6) can be found elsewhere [21,44]. In Equations (5) and (6), stoichiometric concentrations (moles per liter of total emulsion volume) are indicated by brackets, [ ], and the effective or real concentration in a particular region (expressed in moles per liter of the volume of the particular region) are indicated with parentheses, ( ). The subscript T stands for the stoichiometric or total concentration, and the subscripts O, I and W indicate the oil, interfacial and aqueous regions, respectively; Φ_I_ = V_surf_/V_emulsion_ is the surfactant volume fraction (which is equal to that of the interfacial region), and Φ_O_ is the oil volume fraction (Φ_O_ = V_oil_/V_Total_).

(5) $$P_{O}^{I}=\frac{{(\mathrm{AO}}_{I})}{{(\mathrm{AO}}_{O})}$$

(6) $$k_{\mathrm{obs}}=\frac{k_{I}{[\mathrm{AO}}_{T}]P_{O}^{I}}{\Phi_{I}P_{O}^{I}+\Phi_{O}}$$

(7) $$\frac{1}{k_{\mathrm{obs}}}=\frac{\Phi_{O}}{k_{I}{[\mathrm{AO}}_{T}{]P}_{O}^{I}}+\frac{1}{k_{I}{[\mathrm{AO}}_{T}]}\Phi_{I}$$

Equation (7) is the reverse of Equation (6) and predicts that, at fixed [AO_T_] and Φ_O_, the variation in 1/*k*_obs_ with Φ_I_ should be linear, allowing the *P*_O_^I^ and *k*_I_ values to be obtained.

The mechanism of the reaction between ArN_2_^+^ ions and phenols is known, and although details are not strictly necessary for determining the distribution of the antioxidant, it is worth noting that the observed rate constant *k*_obs_ depends on the acidity because the reactive species is the deprotonated form of the phenol (i.e., δ-TOC^−^) [55]. δ-TOC remains neutral at pH < 12 because the p*K*a of δ-TOC has been reported to be 12–14 in cationic micelles, suggesting that δ-TOC exists predominantly in the unionized form [56]. However, as indicated in Introduction Section, charged interfaces modify the local proton concentration, and for this reason, the aqueous pH of the emulsions had to be adjusted to obtain convenient reaction times (in the order of several tens of minutes) [13]. Thus, anionic SDS emulsions (which increase the interfacial acidity) were prepared at pH = 8, while for the neutral and/or anionic ones (prepared with CTAB, TW20 or their mixtures), the aqueous pH was adjusted to pH 3.5. It is also worth noting that, because δ-TOC is not ionized in the pH 3.5–8 range, no acidity effects on the distribution of δ-TOC are expected, in line with results in previous reports [57]. We, however, wanted to make sure that acidity has no effect on the distribution, and, for comparative purposes, we ran some kinetic experiments in emulsions prepared at pH = 4.5.

### 2.5. Determining k_obs_ Values for the Reaction between 16-ArN_2_^+^ and δ-TOC in Intact Emulsions

Equations (6) and (7) establish the relationship between the observed rate and partition constants. Partitioning experiments are usually carried out at constant [AO_T_] and Φ_O_ = V_oil_/V_emulsion_ so that the *P*_O_^I^ values can be obtained from the experimental variation in 1/*k*_obs_ vs. Φ_I_.

**Figure 4:**
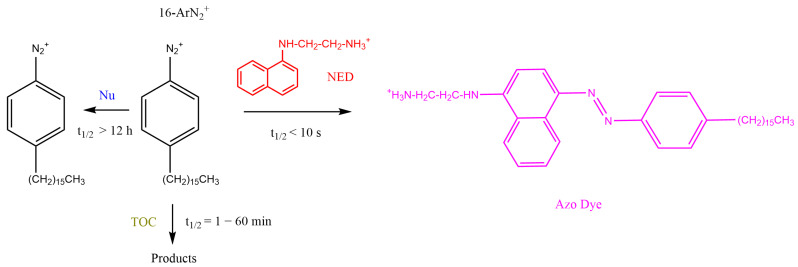
Competitive reactions of 16-ArN_2_^+^ with AOs and with the coupling reagent NED to yield a stable azo dye. Experimental conditions were optimized so that the reaction with NED would be much faster than the spontaneous decomposition and much faster than that with AOs, allowing the monitoring of the disappearance of 16-ArN_2_^+^ with time in emulsions. Further details on the method can be found elsewhere [44,45,58].

Emulsions are opaque, and thus, *k*_obs_ values were obtained by employing a well-established derivatization method that exploits the rapid reaction between the coupling agent N-(1-naphthyl)ethylenediamine, NED, and the chemical probe, 4-hexadecylbenzenediazonium, 16-ArN_2_^+^, as shown in Figure 4. Details of the method, as well as its advantages and limitations, can be found elsewhere [44,45,58].

**Figure 5:**
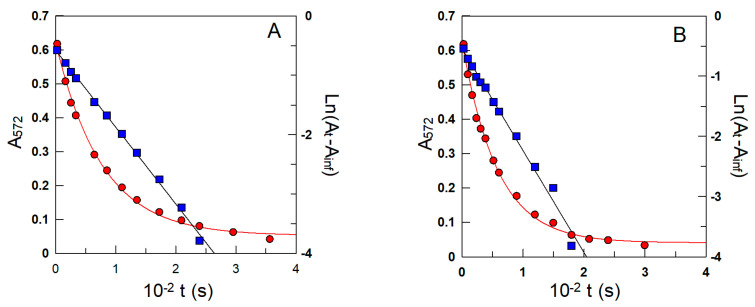
Representative variations in the pseudo-first-order decrease in the absorbance of the azo dye (λ = 572 nm) with time (Figure 4) obtained in intact 1:9 soybean oil/TW20/acidic water (**A**) and oil/CTAB/acidic water (**B**) emulsions. *k*_obs_ values for the reaction between 16-ArN_2_^+^ and the antioxidant were indirectly determined from both the exponential and linear variations in the absorbance with time. The solid lines are the theoretical curves obtained by fitting the (A, t) and (ln(A_t_ −A_inf_), t) pairs of data to the integrated and linearized pseudo-first-order equations. Runs were carried out in the intact emulsions in triplicate, and the average values (deviations less than 7%) were used in calculations. Other experimental conditions: pH = 4.5; Φ_I_ = 0.005; [16-ArN_2_^+^] = 3.0 × 10^−4^ M; [δ-TOC] = 4.0 × 10^−3^ M; T = 25 °C.

Experimental conditions were optimized so that the reaction with NED would be much faster (half-life t_1/2_ < 10 s) than that with the antioxidant (t_1/2_ > 300 s). Briefly, a freshly prepared emulsion (10 mL) was transferred to a thermostated cell. Once the emulsion was thermostated, the reaction between the antioxidant and the probe molecule was initiated by adding an aliquot (16 μL) of a 0.17 M stock 16-ArN_2_^+^ solution in acetonitrile under pseudo-first-order conditions ([AO] >>> [16-ArN_2_^+^]). Independently, 15 numbered and stoppered test tubes were placed in a thermostatic bath (T = 25 °C), and 2.5 mL of a 0.02 M EtOH-BuOH (1:1, *v*/*v*) solution of NED was added to each test tube and allowed to reach thermal equilibrium. Aliquots (200 µL) of the reaction mixtures were removed at specific time intervals and added immediately to test tubes to initiate azo dye formation. Auxiliary experiments showed that the absorbance of the formed azo dye can be linearly correlated with [16-ArN_2_^+^], and therefore, the variation in the absorbance of the azo dye with time can be used to indirectly determine *k*_obs_ by fitting the data to the integrated first-order equation, as illustrated in Figure 5. Duplicate or triplicate experiments gave *k*_obs_ values with deviations lower than 7%. Further experimental details and the advantages and limitations of the method can be found elsewhere [44,46].

### 2.6. Determining Antioxidant Distributions and Effective Concentrations

Kinetic experiments were usually carried out at constant [AO_T_] and Φ_O_ = V_oil_/V_emulsion_ so that the *P*_O_^I^ values could be obtained from the experimental variation in 1/*k*_obs_ vs. Φ_I_, as described in Equation (7) (which is the reciprocal form of Equation (6), and this predicts that the plots should be linear with positive intercepts. Once the *P*_O_^I^ values are known, the percentages and effective concentrations of the AOs in the interfacial and oil regions can be determined by employing Equations (8)–(11). Details on these calculations, as well as the equations for antioxidants with moderate and low hydrophobicity, can be found elsewhere [46,52,53,54,58,59].

(8) $${\%\mathrm{AO}}_{I}=\frac{100P_{O}^{I}\Phi_{I}}{\Phi_{I}P_{O}^{I}{+\Phi }_{O}}$$

(9) $${\%\mathrm{AO}}_{O}=\frac{{100\Phi }_{O}}{\Phi_{I}P_{O}^{I}{+\Phi }_{O}}$$

(10) $${(\mathrm{AO}}_{I})=\frac{{[\mathrm{AO}}_{T}{](\%\mathrm{AO}}_{I})}{\Phi_{I}}$$

(11) $${(\mathrm{AO}}_{O})=\frac{{[\mathrm{AO}}_{T}{](\%\mathrm{AO}}_{O})}{\Phi_{O}}$$

### 2.7. Oxidative Stability of Emulsions: Schaal Method

Emulsions were allowed to spontaneously oxidize at T = 60 °C in the dark, and the progress of the oxidation reaction was assessed as in previous works [60,61,62,63,64,65] by monitoring the formation of primary oxidation products with time (AOCS Official Method Ti 1a 64). Aliquots (25 µL) of the emulsions were removed at selected times and diluted to 10 mL with 2-propanol, and the absorbance was determined at λ = 233 nm. Emulsions with no added antioxidant were used as the control, and the relative efficiency of the antioxidant was assessed by comparing the time needed to observe an increase of 0.5% in the formation of conjugated dienes. Experiments were carried out in triplicate, and only the average values are reported.

## 3. Results and Discussion

### 3.1. Determining the Partition Constants of δ-TOC in the Oil–Interfacial, P_O_^I^, Region in Intact Soybean Oil Emulsions

**Figure 6:**
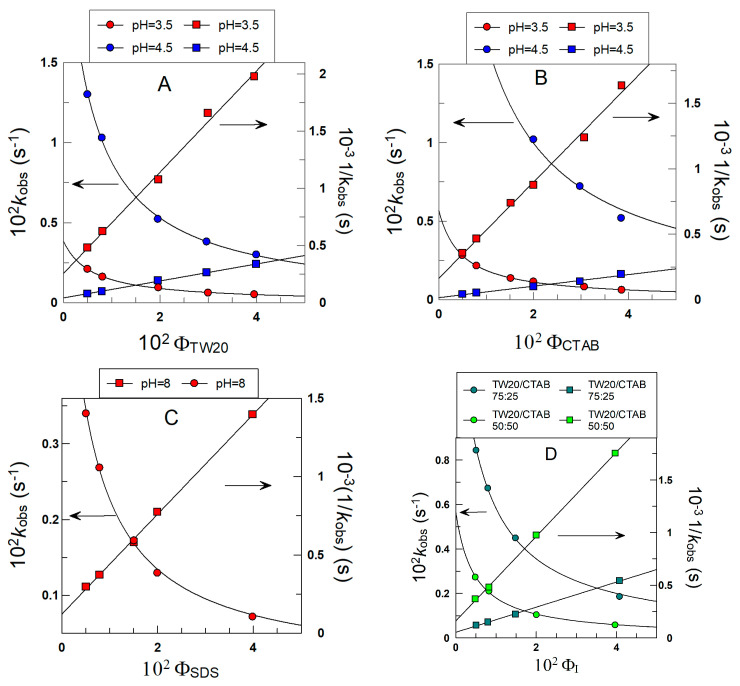
Variations in the observed rate constant *k_obs_* between the chemical probe and δ-TOC with the surfactant volume fraction Φ_I_ in intact emulsions prepared with different surfactants (TW20, CTAB and SDS). The solid lines are the theoretical curves obtained by fitting experimental pairs of data (*k_obs_*, Φ_I_) to Equations (6) and (7). Experimental conditions: emulsions 1:9 (o/w, *v*/*v*); T = 25 °C; [δ-TOC] = 4 × 10^−3^ M.

Figure 6 shows the variations in *k*_obs_ with Φ_I_ for the reaction of 16ArN_2_^+^ with δ-TOC in intact 1:69 (o/w) soybean oil emulsions at T = 25 °C prepared with different surfactants. In all runs, *k*_obs_ values decrease asymptotically 4–6-fold upon increasing the surfactant volume fraction from Φ_I_ = 0.005 to Φ_I_ = 0.04.

Figure 6A–D also show the effects of the electrical nature of the surfactant on *k*_obs_. At a given surfactant volume fraction Φ_I_, *k*_obs_ values increase 6–9-fold upon increasing the pH of the aqueous phase as a consequence of the increase in the ionized form of the antioxidant, which is the reactive species, as indicated in Section 2.4.

The excellent fits of the (*k*_obs_ Φ_I_) pairs of data to Equations (6) and (7) indicate that the assumptions of the pseudophase kinetic model are fulfilled, and the partition constants *P*_O_^I^ and the intrinsic rate constants *k*_I_, obtained from the slopes and intercepts of the linear fits, are compiled in Table 1.

The picture that arises from the results in Table 1 indicates that the partition constants *P*_O_^I^ of δ-TOC are independent of the nature and/or charge of the surfactant employed in the preparation of the emulsions (differences less than 20%), with an average value of *P*_O_^I^ = 20 ± 4. The results in Table 1 also show the effects of acidity on the reaction between 16-ArN_2_^+^ and δ-TOC. At a given pH (e.g., 3.5), the rate constants *k*_I_ are essentially the same, indicating that the medium composition is similar in all cases; that is, the nature of the surfactant does not affect the reaction. However, an increase in pH from 3.5 to 4.5 to 8 (SDS emulsions) causes *k*_I_ to increase (for example, in TW20 emulsions, *k*_I_ increases from 0.57 to 3.37), in line with the acid dependence of the reaction (see Section 2.4 for further details).

**Figure 7:**
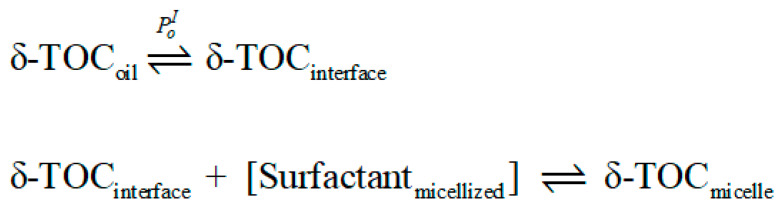
Representation of the equilibrium between the antioxidant located in the interfacial–oil regions of emulsions and the micelles present in the aqueous phase of the emulsions ([surfactant]_micellized_).

It may be worth noting here that the results reflected in Table 1 are in line with those recently reported by Fernández-Ventoso et al. [57], who investigated the effects of acidity on the distribution of tocopherols in olive and soybean oil emulsions, finding that a change in acidity does not change the distribution of tocopherols but modifies the intrinsic rate constants *k*_I_. However, these results apparently contradict those recently reported by Ichingolo et al. [66], who investigated the role of SDS on the apparent distribution of TOC in soybean emulsions, reporting that an increase in the surfactant concentration increases the amount of SDS micelles in the aqueous phase and that the amount of TOC in the “aqueous” phase increases. They claimed that the solubility of TOC in water is not affected by the presence of SDS molecules, but after reaching a certain concentration, called the critical micelle concentration, SDS molecules self-associate and form micelles, which can solubilize TOC molecules. Thus, an increase in the concentration of micelles would imply that the amount of TOC associated with the micelles increases according to the equilibria shown in Figure 7.

We think that the reasons for the discrepancy lie in the fact that the determinations of the amount of TOC in the aqueous phase made by Ichingolo et al. [66] were performed after breaking down the emulsions, and therefore, their results may be biased because of the disruption of the existing equilibria. The results in Table 1, in contrast, were obtained in intact emulsions. The variations in *k*_obs_-Φ_I_ shown suggest that the partition constants of TOC are not altered by the presence of increasing amounts of surfactants. Otherwise, if the partition constants *P*_O_^I^ depend strongly on the concentration of the surfactant employed [surfactant] (which would change the concentration of micelles in the aqueous phase), we should obtain random variations in the *k*_obs_-Φ_I_ profiles, which would imply large deviations from linearity in the *k*_obs_ vs. Φ_I_ plots, which is not the case. We note that, according to McClements [4,67] and others [21,44], micelles and emulsions are dynamic structures, breaking down and reforming at diffusion-controlled rates. Thus, no distinction between interfaces can be made, and the location of the antioxidant should be considered as an average position. The effect of increasing [surfactant] is an increase in the interfacial volumes of all physical structures present in the system and hence a decrease in the effective concentration of the antioxidant in the interfacial region.

### 3.2. Distribution and Interfacial Concentrations of δ-TOC

**Figure 8:**
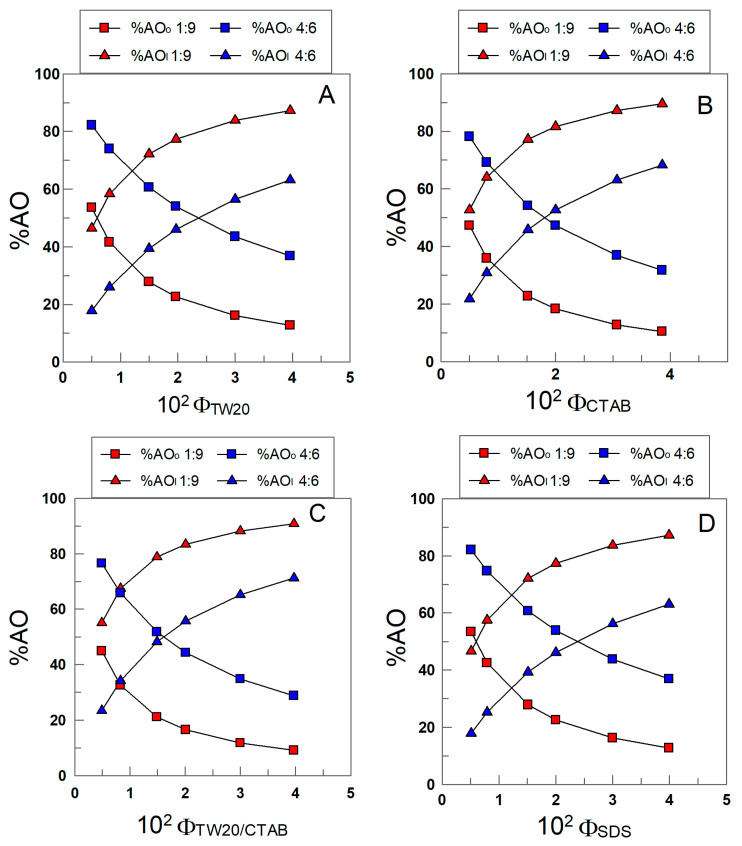
Variations in the percentage of TOC in the oil (squares) and interfacial region (triangles) with the surfactant volume fraction and the oil–water ratio in 1:9 and 4:6 (o/w, *v*/*v*) soybean emulsions prepared with TW20 (**A**), CTAB (**B**), TW20/CTAB (1:1, (**C**)) and SDS (**D**).

The distribution of δ-TOC was determined by employing Equations (8) and (9). Figure 8A–D show the variations in %δ-TOC with Φ_I_ in the different emulsions. In all cases, more than 45% of δ-TOC is located in the interfacial region of 1:9 (o/w, *v*/*v*) emulsions, and the percentage increases upon increasing Φ_I_. At any given Φ_I_ value, the results in Figure 8A–D show that the percentage of δ-TOC in the oil or interfacial region is essentially the same independently of the electrical charge of the surfactant; for example, when Φ_I_ = 0.005 (the lowest surfactant concentration employed), %TOC_I_ ≈ 45 in all emulsions (differences less than 10%), and when Φ_I_ = 0.04, %δ-TOC_I_ ≈ 90.

The results in Figure 8A–D show the effects of changing the oil-to-water ratio (emulsions 1:9 and 4:6). An increase in the percentage of oil in the emulsion decreases %δ-TOC_I_ to similar extents independently of the electrical charge of the surfactant. For example, in TW20 emulsions, %δ-TOC_I_ decreases from 55 to 23 (Φ_I_ = 0.005) upon switching from 1:9 to 4:6 emulsions, while in SDS emulsions, %δ-TOC_I_ decreases from 47% to 18%.

The effective concentration of the antioxidant in the interfacial region plays a central role in determining the relative efficiency of the antioxidant because, for an antioxidant to be effective in emulsions, the rate of the inhibition reaction between the antioxidant and the peroxyl radicals must be higher than the rate of propagation of the lipid oxidation reaction. The rate of the inhibition reaction depends, among others, on the effective concentration of the antioxidant in the interfacial region. Thus, we determined the effective concentrations from the distribution data by employing Equations (10) and (11).

**Figure 9:**
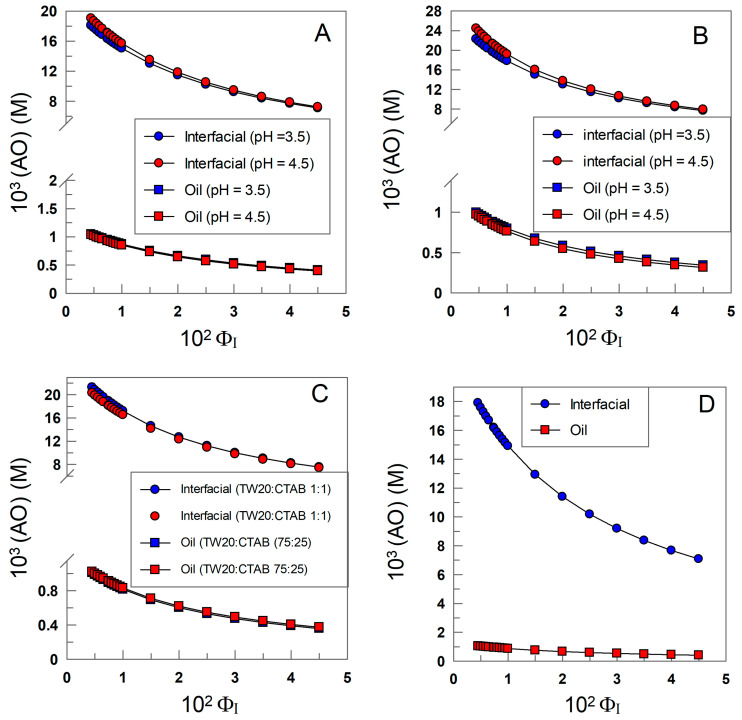
Variations in the effective concentration of δ-TOC in the oil (squares) and interfacial region (triangles) with the surfactant volume fraction in 4:6 (o/w, *v*/*v*) soybean emulsions prepared with TW20 (**A**), CTAB (**B**), TW20/CTAB (1:1 and 75:25, (**C**)) and SDS (**D**); [TOC_T_] = 5 × 10^−4^ M.

Figure 9A–D show that the effective concentration of δ-TOC in any of the emulsions is much higher, 10–100-fold, than the stoichiometric concentration and decreases upon increasing the surfactant volume fraction Φ_I_. It may be surprising at first glance that, in any of the investigated emulsions, (AO_I_) decreases upon increasing Φ_I_ in spite of the fact that Figure 8A–D show that the %AO_I_ increases upon increasing Φ_I_. These apparently contradictory results can be easily rationalized when considering the opposite effects that cause an increase in the surfactant volume fraction. On one side, an increase in Φ_I_ increases the fraction of the antioxidant in the interfacial region, but at the same time, it also increases the interfacial volume so that the net effect is a dilution of the antioxidant. For instance, in SDS emulsions, an increase in Φ_I_ from 0.005 to 0.045 (i.e., a 9-fold increase in the interfacial volume) leads to an increase in %AO_I_ of only 2-fold (from ~45 to 90), Figure 8, leading to the effective dilution of the antioxidant.

**Figure 10:**
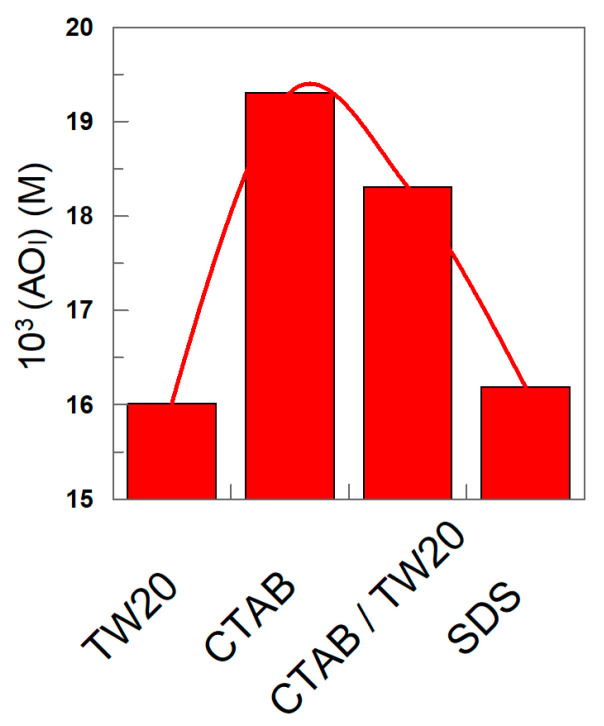
Variation in the effective interfacial concentration (M) of δ-TOC in emulsions prepared with different surfactants (TW 20, CTAB, CTAB/TW20 1:1 and SDS). Experimental conditions: 4:6 (o/w, *v*/*v*) emulsions; Φ_I_ = 0.008; [δ-TOC_T_] = 5 × 10^−4^ M.

The nature of the surfactant seems to have a modest, though significant, effect on the effective concentration of the antioxidant, as shown in Figure 10. For instance, at Φ_I_ = 0.0045, the highest interfacial concentration is found for the emulsions prepared with CTAB, while slightly lower values (~20%) are found for those prepared with TW20 and SDS.

In previous papers, we demonstrated that the effective interfacial concentration is one of the main parameters controlling the efficiency of antioxidants in emulsions, finding that, under some circumstances, the efficiency of potent antioxidants in bulk solutions diminishes substantially in emulsions because their effective concentrations are not high enough to make the rate of the inhibition reaction higher than that of the propagation step [21,22,68]. Thus, changes in the effective concentration of the antioxidant, though modest, may have a significant effect on the efficiency of δ-TOC in inhibiting lipid oxidation. Thus, it is important to analyze the oxidative stability of emulsions in both the presence and absence of the antioxidant.

### 3.3. Oxidative Stability of Emulsions: Effects of the Interfacial Charge

**Figure 11:**
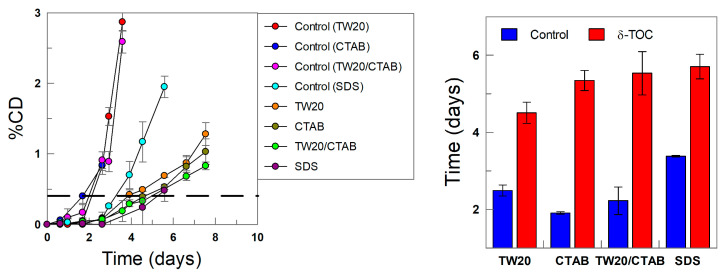
(**Left**) Kinetics of production of conjugated dienes with time in 4:6 (o/w, *v*/*v*) soybean emulsions at T = 60 °C in the absence (control) and presence of δ-TOC prepared with different surfactants (TW20, CTAB, CTAB/TW20 1:1 and SDS), Φ_I_ =0.008 and [δ-TOC_T_] = 5 × 10^−4^ M. (**Right**) The relative oxidative stability assessed by determining the time required to increase the percentage of CDs in ΔCD = 0.5% (dashed line in kinetic profiles).

The oxidative stability of the emulsions was assessed in both the presence and absence of the antioxidant by monitoring the variation in the formation of conjugated dienes with time, as shown in Figure 11. The kinetic profiles are biphasic, showing an initially slow production of CDs (called the induction period or lag time), followed by a second stage, where the production of CDs is much faster. The latter corresponds to the uninhibited reaction, which is attained after the consumption of most of the added antioxidant.

The results in Figure 11 show that, in the absence of the antioxidant (control experiments), the relative oxidative stability of the emulsions, as measured in terms of the time required to reach an increase in the production of CDs of 0.5%, follows the order CTAB < TW20 ~ TW20/CTAB < SDS. This percentage was chosen so that all emulsions would undergo the induction period while being low enough to minimize the decomposition of CDs to secondary oxidation products. The results in Figure 11 show that the electrical nature of the surfactants seems to play a role in the oxidative stability of the emulsions. In our experiments, we employed stripped oils and deionized water (see experimental section); hence, it is not likely that such an effect can be attributed to the potential presence of prooxidant metals in the system, but the results are consistent with literature reports on the role of the interfacial charge and the nature of the surfactants employed in lipid oxidation reactions.

Lee et al. [69] investigated the influence of blends of surfactants on lipid oxidation, reporting that SDS prevents TBARS generation, retarding the oxidation of the emulsions. Yi et al. [8] analyzed the effects of emulsifier charges on the oxidative stability of corn oil-in-water emulsions, concluding that cationic emulsions had lower oxidative stability, followed by samples with anionic and then neutral surfactants. They reported that the prooxidative properties of CTAB in o/w emulsions could be due to the acceleration of hydroperoxides by CTAB and/or the prooxidative properties of bromide ions. Kasaikina et al. [17,70] investigated the role of surfactants in the kinetics of lipid oxidation and reported that the presence of cationic surfactants (e.g., CTAB) increases the initiation rate, resulting in the overall acceleration of oxidation, which suggests that the prooxidative action results from the formation of mixed hydroperoxide–surfactant micelles, favoring the decomposition of hydroperoxides, while anionic surfactants (e.g., SDS) partially hydrolyze to dodecyl alcohol, inhibiting the oxidation reaction, as reflected in a decrease in the induction periods. For example, the oxidation of sunflower triglycerides was accelerated by cationic surfactants (CTAB, CTACl) but was not affected by SDS. Reactions were run by employing different substrates, and details on the kinetics of the reactions under different experimental conditions can be found elsewhere [17].

**Figure 12:**
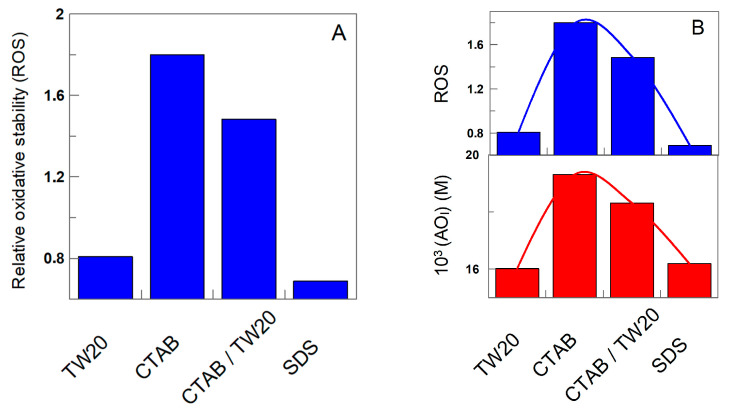
(**A**) Relative oxidative stability of charged emulsions, as determined by Equation (12). (**B**) Correlation between the relative oxidative stability and the effective concentration of δ-TOC in the interfacial region of the emulsions.

The exact role of surfactants in the kinetics of lipid oxidation is not, however, conclusive, and to make the relative oxidative stability independent of the matrix effect, we determined the relative oxidative stability, ROS, in the presence of δ-TOC by employing Equation (12), where t_δ-TOC_ and t_control_ are the times necessary to obtain an increase in the production of CDs of 0.5%, and the results are displayed in Figure 12.

(12) $$\mathrm{ROS}=\frac{t_{\delta -\mathrm{TOC}}-t_{\mathrm{control}}}{t_{\mathrm{control}}}$$

The results may appear surprising, because Figure 11 and Figure 12 clearly show that the addition of δ-TOC has a significant effect on the oxidative stability of the emulsions, totally the opposite to that found in its absence (Figure 11): emulsions prepared with CTAB are the most chemically stable, while the less stable are those prepared with SDS. The relative oxidative stability in the presence of δ-TOC follows the order SDS ≈ TW20 << TW20/CTAB < CTAB. However, the results can be easily rationalized when comparing the relative oxidative stability with the effective interfacial concentrations of the antioxidant: the relative oxidative stability correlates pretty well with the effective interfacial concentration, as shown in Figure 11 (Right), highlighting three important features:

(1) The acidity of the interfacial region does not affect the oxidative stability or the distribution of δ-TOC;
(2) The effective interfacial concentration is a crucial parameter that controls the oxidative stability of emulsions;
(3) There exists a direct relationship between the effective concentration of the antioxidant in the interfacial region and the oxidative stability.

## 4. Conclusions

Lipid oxidation in oil-in-water emulsions can be affected in different ways by the charge of the emulsions [13,20,32]. Such effects mostly arise from the electrostatic forces involved, which may significantly change the effective concentrations of metallic prooxidants and the acidity of the interfacial region, affecting the ionization of antioxidants and their location and, hence, their effective concentration. The results shown here demonstrate that there is no need to argue the potential role of micelles in the aqueous phase or, even less, treat them as separate physical structures, where antioxidants bind with different association constants other than those in emulsions. These results, combined with those reported for nonionic and ionic emulsions, demonstrate that pseudophase kinetic models provide general, coherent explanations of chemical reactivity in homogeneous micelles, microemulsions, vesicles and emulsions and with all types of basic surfactant structures: nonionic, cationic, anionic and zwitterionic [13,39].

The results also suggest that (i) tuning the rates of acid-sensitive reactions can be accomplished by selecting the right salt concentration and anion types, which may be useful for promoting or inhibiting reactions, and (ii) selecting the appropriate medium/conditions is important for the stabilization of bioactive molecules in interfaces, whose kinetics of degradation significantly depends on the pH of the medium.

Finally, it is worth noting that the results reported here should aid in interpreting the effects of ionic surfactants on chemical reactivity in emulsions in general and in selecting the most efficient antioxidant for a particular food application by choosing conditions that maximize its effective concentration in the interfacial region of the emulsion. The use of antioxidants with high radical-scavenging capacity is crucial to inhibit lipid oxidation, but their accumulation at the interface of emulsions is also key to modulating their efficiency in inhibiting lipid oxidation. For instance, chlorogenic antioxidants do not show a significant effect on inhibiting lipid oxidation in emulsions because their interfacial concentrations may not be high enough to make the rate of the inhibition reaction faster than the rate of radical propagation. While many papers report on the ability of phenolics to inhibit lipid oxidation in both experimental model systems and foods, the role of the interfacial concentration is frequently neglected, and much more needs to be understood on how different parameters affect the local concentrations of reactants and structure–activity relationships of antioxidants.

## Figures and Tables

**Table 1:** Values of the ξ potential, partition constant *P*_O_^I^ and rate constant in the interfacial region *k*_I_ in the different emulsions employed.

Surfactant	HLB	pH	ξ (mV)	*P*_O_^I^	10^2^ *k*_I_ (M^−1^ s^−1^)
TW20	16.7	3.5	−2.0	17.3 ± 4.0	0.57 ± 0.03
		4.5	---	18.4 ± 1.3	3.37 ± 0.07
CTAB	15.8	3.5	58.7	22.3 ± 6.7	0.68 ± 0.03
		4.5	---	25.1 ± 7.6	5.67 ± 0.34
SDS	40.0	8	−74.5	17.1 ± 3.3	0.78 ± 0.02
TW20/CTAB (50:50)	16.3	3.5	31.8	21.1 ± 2.2	0.62 ± 0.01
TW20/CTAB (75:25)	16.5	3.5	44.5	19.9 ± 2.3	0.59 ± 0.04
